# Benefits and challenges to therapeutic targeting of bile acid circulation in cholestatic liver disease

**DOI:** 10.1097/HEP.0000000000001438

**Published:** 2025-07-02

**Authors:** Michael Trauner, Saul J. Karpen, Paul A. Dawson

**Affiliations:** 1 Department of Internal Medicine III, Division of Gastroenterology and Hepatology, Medical University of Vienna, Vienna, Austria; 2 Stravitz-Sanyal Institute for Liver Disease and Metabolic Health, Virginia Commonwealth University School of Medicine, Richmond, Virginia, USA; 3 Department of Pediatrics, Emory University School of Medicine and Children’s Healthcare of Atlanta, Atlanta, Georgia, USA

**Keywords:** enterohepatic circulation, ileum, nuclear receptor, proximal renal tubule, transport

## Abstract

Progress in our understanding of the molecular basis of bile acid (BA) transport in the liver, bile ducts, intestine, and kidney has not only advanced our understanding of the pathophysiology of cholestasis and metabolic dysfunction-associated liver disease but also led to novel therapeutic approaches targeting BA transport and signaling within the entero-nephro-hepatic circulation. This includes BA transport modulators such as inhibitors of the apical BA-transport system in the terminal ileum and proximal renal tubule (IBAT/ASBT inhibitors) and basolateral (sinusoidal) BA uptake in hepatocytes (NTCP inhibitors). In addition to altering membrane transporter function by targeting IBAT/ASBT and NTCP, there is an array of potentially additive therapeutic approaches which include receptor agonists acting via nuclear receptor (FXR, PPAR)-mediated transcriptional modification of BA synthesis and transport genes and BA analogs such as norucholic acid (previously known as norUDCA) that undergo cholehepatic shunting. This article reviews established and emerging molecular and clinical rationales for therapeutic targeting of BA circulation and signaling in liver diseases with a specific focus on cholestatic disorders.

## INTRODUCTION

**FIGURE 1:**
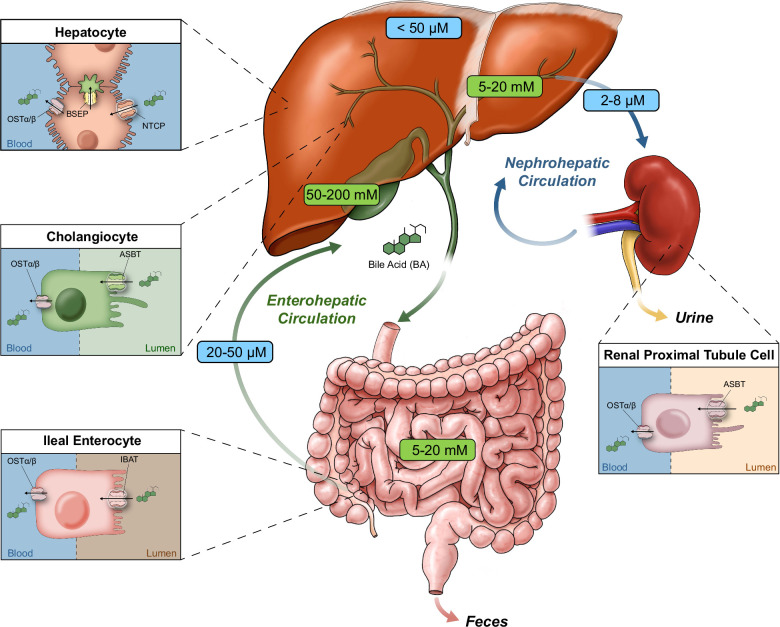
The entero-nephro-hepatic circulation of BAs in health. The central portion of the figure graphically outlines the key organ and tissue contributions to the transport of BAs. Note the range of BA concentrations in the different fluids and tissues. Callout images highlight the major BA transporters in hepatocytes, cholangiocytes, ileal enterocytes, and proximal renal tubules. Note that current convention labels the ileal BA transporter as IBAT and the other sites as ASBT, but note that these are the same protein (SLC10A2). Portions of the figure are adapted from Fickert and Wagner.^3^ Abbreviations: ASBT, apical sodium-dependent BA transporter; BAs, bile acids; BSEP, bile salt export pump; IBAT, ileal BA transporter; NTCP, Na+taurocholate cotransporting polypeptide; OSTα/β, organic solute transporter α/β.

**FIGURE 2:**
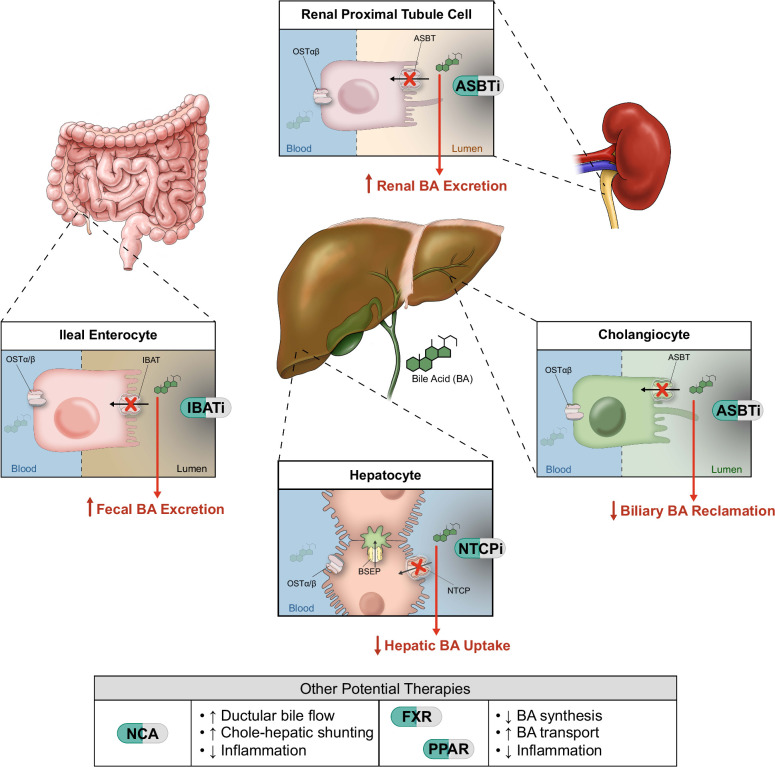
Principal therapeutic targets within the entero-nephro-hepatic circulation of BAs in cholestasis. The increased accretion of BAs in the cholestatic liver, along with increased serum concentrations, leads to an expected enhanced exposure of the apical membrane of the proximal tubule after glomerular filtration. As opposed to the non-cholestatic state (Figure 1), where there is limited exposure of the renal IBAT/ASBT to BAs, given the increased serum BA concentrations, there is significant renal reclamation via ASBT in the proximal renal tubules and re-presentation to hepatic NTCP for re-uptake. Current knowledge identifies non-redundant opportunities to interfere with BA reclamation in various compartments. A gut-restricted IBAT inhibitor (IBATi) will interfere with luminal BA uptake, leading to increased fecal BA elimination. An NTCP inhibitor (NTCPi) will lead to reduced hepatic BA accumulation and elevated serum BA levels. A systemic IBAT/ASBT inhibitor (ASBTi) will primarily block renal BA reclamation (although it may also target the cholangiocyte and ileum), leading to increased BA excretion in the urine. Each of these approaches, in preclinical models, has been shown to markedly attenuate the hepatic consequences of cholestasis, while the systemic ASBTi has also been shown to ameliorate cholemic nephropathy (also see text for further details). In addition to the BA transporter inhibitors, NCA, and agonists target the nuclear receptor regulators FXR and PPARs have been shown in preclinical and clinical studies to attenuate cholestasis and hepatic inflammation, via variable mechanisms. Note that current convention labels the ileal BA transporter as IBAT and the other sites as ASBT, but these are the same protein (SLC10A2). Abbreviations: ASBT, apical sodium-dependent BA transporter; BAs, bile acids; BSEP, bile salt export pump; FXR, farnesoid X receptor; IBAT, ileal BA transporter; NCA, norucholic acid; NTCP, Na+-taurocholate cotransporting polypeptide; OSTα/β, organic solute transporter α/β; PPARs, peroxisome proliferator-activated receptors.

Advances over the past 2 decades in our understanding of the molecular basis for bile acid (BA) cycling and signaling (Figure 1)^1–4^ have led to a range of novel therapeutics which have now entered the phase of clinical application. These BA-targeting therapeutics can be broadly categorized as “BA Transport Modulators” and “Receptor Agonists” (Figure 2). BA Transport Modulators act by changing the concentration or composition of the *endogenous* BA pool in compartments of the body, thereby reducing BA cytotoxicity and altering the signaling properties of the BA pool. The recently developed therapeutic modulators of BA transport include small-molecule inhibitors of the ileal BA transporter (IBAT, also known as the apical sodium-dependent BA transporter ASBT; *SLC10A2*) and a peptide-based inhibitor of the structurally related liver Na^+-^taurocholate cotransporting polypeptide (NTCP; *SLC10A1*). Inhibition of these transporters reduces BA return to the liver, thereby limiting hepatic BA accumulation under cholestatic conditions. Moreover, altering transport and thereby BA availability/distribution also affects BA signaling through dedicated nuclear and G-protein coupled receptors in the gut–liver axis and other sites, and is part of the therapeutic mechanism of action of BA Transport Modulators. Receptor agonists include *exogenous* small molecules and mimetics that directly target the nuclear BA receptor/farnesoid X receptor (FXR; *NR1H4*) as well as other nuclear (hormone) receptors (eg, various isoforms of peroxisome proliferator-activated receptor; PPAR), and the downstream regulator/intestinal hormone fibroblast growth factor 19 (FGF19). The receptor agonists impact BA homeostasis and cycling in addition to directly stimulating hepatoprotective mechanisms. In addition to the recently approved therapies, new approaches are under development that include “correctors” to restore hepatobiliary transporter function for primary genetic forms of cholestasis, new BA transporter modulators with expanded target tissue specificity, and new BA analogs that act to improve hepatic bile secretion and BA circulation. This concise review provides an overview of the molecular basis/rationale, mechanisms of action, and clinical efficacy of novel and emerging therapies targeting the BA circulation in cholestatic liver diseases, with a focus on adult cholestatic disorders (for discussion of pediatric cholestatic disorders, see related article in this series^5^).

## PHYSIOLOGY OF BA TRANSPORT AND HOMEOSTASIS

The major anatomic components of the BA circulation include the liver, biliary tract, gallbladder, small intestine, colon, kidney, portal venous circulation, and systemic circulation. Classically termed the enterohepatic circulation to reflect the intestine and liver’s central role in BA cycling, it is important to recognize that BAs are also actively transported/reabsorbed by the kidney in health and disease. Under normal physiological conditions, specific membrane carriers in the liver, intestine, and kidney function to quantitatively transport BAs, and this efficient intestinal/renal BA reabsorption and hepatic BA extraction/secretion enable an effective recycling and conservation mechanism that largely restricts BAs to the intestinal and hepatobiliary compartments. The major cell types (hepatocyte, ileal enterocyte, renal proximal tubule cell) and transporters responsible for the “​​​​​*Enteronephrohepatic circulation*” of BAs in humans are summarized in Figure 1. In hepatocytes, BAs are taken up across the sinusoidal membrane by a sodium-dependent transport mechanism via the NTCP and a sodium-independent mechanism via members of the Organic anion transport polypetide (OATP) family, which include OATP1B1/*SLCO1B1* and OATP1B3/*SLCO1B3* in humans. Following uptake, BAs are secreted across the canalicular membrane into bile by the bile salt export pump (BSEP; *ABCB11*), which preferentially transports conjugated (amidated) versus unconjugated BAs.^6^ Besides being conjugated to glycine/taurine, BAs can undergo additional phase I metabolism/hydroxylation by cytochrome P450 enzymes (eg, CYP3A4^7^) and phase II metabolism/sulfation (eg, SULT2A1^8^) and glucuronidation (eg, UGT1A3, UGT2B7^9,10^). Although these are minor pathways under normal physiological conditions, hepatic phase I/II metabolism of BAs is induced as an adaptive response to cholestasis.^11^ If canalicular secretion is intact, polyhydroxylated BAs are secreted into bile by the multidrug resistance-associated protein-2 (MRP2; *ABCC2*), P-glycoprotein (MDR1; *ABCB1*), and BSEP,^12^ whereas sulfated and glucuronidated BAs are secreted into bile primarily by MRP2.

After their secretion into bile, BAs enter the gallbladder for temporary storage or pass directly into the lumen of the small intestine. There, a minor fraction of the BAs is reclaimed by passive absorption down the length of the small intestine and colon, whereas most conjugated BAs are efficiently transported by IBAT into ileal enterocytes. After uptake, BAs bind the cytosolic Ileal BA binding protein (IBABP; *FABP6*), and then are exported across the basolateral membrane by the heteromeric transporter, OSTα/β (*SLC51A*, *SLC51B*).^2^ Following intestinal absorption, BAs return via the portal circulation to the liver for uptake and resecretion into bile.^13^ BAs that escape hepatic first pass clearance enter the systemic circulation, where the non-protein-bound BA fraction undergoes glomerular filtration. Rather than be excreted in urine, BAs are almost quantitatively reabsorbed from the kidney proximal tubules by IBAT and OSTα/β and sent back to the liver for uptake/resecretion into bile. Under normal physiological conditions, systemic BA concentrations are low, which limits renal exposure and the kidney’s quantitative contribution to BA cycling.^14,15^ As a result of efficient transport by the intestine, liver, and kidney, most BAs secreted by hepatocytes into bile have previously been synthesized and undergone entero(nephro)hepatic cycling, with <10% being newly synthesized. IBAT and OSTα/β are also expressed by a subset of cholangiocytes lining the biliary tract and by gallbladder epithelial cells.^16,17^ Contrary to IBAT’s role in ileal enterocyte and renal proximal tubule cells, the function of IBAT in cholangiocytes and gallbladder epithelial cells is not quantitative reabsorption of BAs. Currently, it is thought that IBAT and OSTα/β mediate cholehepatic or cholecystohepatic shunting of a small fraction of BAs in bile; however, our understanding of the functional and therapeutic significance of BA transporter expression in the biliary tract and gallbladder epithelium remains the subject of ongoing investigation.^18–20^

BAs control their own synthesis, transport, and metabolism by activating nuclear receptors such as FXR, the pregnane X receptor (PXR, *NR1I2*), constitutive androstane receptor (CAR, *NR1I3*), and vitamin D receptor (VDR, *NR1I1*).^21^ Hepatic BA synthesis from cholesterol is regulated in a negative feedback fashion by FXR in the liver and the ileum. Under normal physiological conditions, the major regulatory pathway involves BA activation of FXR in ileal enterocytes to induce synthesis and secretion of the endocrine polypeptide hormone FGF19 (Fgf15 in mice) into the portal circulation. FGF19 then travels to the liver and signals on hepatocytes via a cell surface complex of FGFR4 and the β-klotho protein to repress CYP7A1 expression and BA synthesis. In addition to the FXR–FGF19–FGFR4 gut–liver signaling pathway, direct BA activation of FXR in hepatocytes induces expression of the orphan nuclear receptor/transcriptional repressor small heterodimer partner (SHP; *NR0B2*), which acts to repress CYP7A1 expression.^22^ In the liver, NTCP expression is also repressed by SHP, whereas expression of the canalicular membrane transporters for phospholipids (multidrug resistance gene 2/3; MDR2/3, *ABCB4*), conjugated organic anions (MRP2), and conjugated BAs (BSEP) is all directly induced by BAs acting through FXR. In addition to transcriptional regulation via nuclear receptors, hepatobiliary transport function is also regulated by posttranscriptional and posttranslational mechanisms, such as transporter protein insertion and retrieval from the membrane and direct protein modifications such as phosphorylation.^23,24^ In the ileum, IBAT expression is negatively regulated by BAs acting through FXR induction of SHP, which interferes with liver receptor homologue 1 (LRH-1; *NR5A2*) activation of IBAT expression. Conversely, BAs act through FXR to coordinately induce expression of IBABP and both subunits of OSTα/β heteromeric transporter to prevent ileal enterocyte BA overload and promote BA homeostasis.^25,26^ In addition to FXR, other nuclear receptors have been shown to induce IBAT expression, including the glucocorticoid receptor (GR; *NR3C1*), PPARα (*NR1C1*), and VDR.^27–29^

## ALTERATIONS OF BA TRANSPORT AND HOMEOSTASIS IN CHOLESTASIS

Cholestasis is characterized by an impairment of hepatobiliary excretory function, resulting in the accumulation of BAs and other biliary constituents in the liver and systemic circulation.^30,31^ This disruption can be complete, for example, due to mechanical obstruction by a stone, dominant stricture or tumor, or a severe genetic defect in a key transporter, membrane structural protein, or molecular chaperone. The changes in hepatobiliary function can also be more subtle such as in the pre-ductopenic stages of cholangiopathies or with incomplete/heterozygous genetic defects, as evidenced by the range of cholestatic phenotypes associated with genetic variants in hepatobiliary membrane transporters, intracellular chaperone proteins (which are important for intracellular trafficking and membrane insertion of those transporters), intercellular tight junction proteins, and direct regulators of key hepatobiliary genes.^32–35^ A clinically relevant example of the broad spectrum of phenotypes associated with hereditary transport defects is MDR3/ABCB4 deficiency, which presents as a continuum of clinical manifestations ranging from mild liver enzyme abnormalities, intrahepatic cholestasis of pregnancy, low phospholipid-associated cholelithiasis (LPAC), and idiopathic ductopenia/biliary fibrosis to cirrhosis and hepatocellular and cholangiocellular cancer.^36^

Considerable progress has been made in understanding not only the molecular basis of normal bile secretion^1,2,23^ but also the consequent pathologies from cholestasis.^30,31^ Transporter alterations driving and responding to cholestasis include (i) primary genetic defects across the spectrum of hereditary cholestasis,^33^ (ii) secondary adaptive, mostly transcriptional changes which can be interpreted as an attempt to counteract retention of cholephiles in the cholestatic liver,^31^ and (iii) secondary transcriptional and posttranscriptional changes that perpetuate the functional impairment of bile secretion and aggravate cholestatic liver injury.^37^ The spectrum of genetic transport defects has been covered by several excellent reviews.^32,33^ As adaptive mechanisms in cholestasis, hepatocyte expression of phase I/II enzymes and basolateral (sinusoidal) membrane BA exporters are induced to detoxify BAs and promote efflux of conjugated BAs and BA metabolites to reduce the hepatocellular BA burden.^38–40^ The transporter changes include mainly transcriptional induction of the hepatocyte basolateral membrane BA exporters MRP3 (*ABCC3*), MRP4 (*ABCC4*), and OSTα/β to redirect BAs into the systemic circulation and partially compensate for impaired canalicular BA excretion into bile via BSEP and MRP2.^31,39,41^ Although downregulation of uptake systems including NTCP and OATP1B1/1B3 in humans^42–44^ (Oatp1a1 in mice^45,46^) can also be seen as “adaptive” to limit the re-uptake of BAs into the cholestatic liver, the downregulation of sinusoidal membrane BA uptake is incomplete. Together with reduced expression/activity of the canalicular efflux pumps (BSEP, MRP2), the changes in NTCP and OATP1B1/1B3 expression are somewhat counterproductive adaptations that maintain the chronic cholestatic state.^31,47^ The functional consequences of transporter expression changes on hepatic BA flux could be directly quantified in cholestatic patients using positron emission tomography and the conjugated BA tracer [N-methyl-^11^C]cholylsarcosine (^11^C-Sar). Those studies demonstrated an increased back-flux of ^11^C-Sar from hepatocytes into blood, which was insufficient to compensate for reduced secretion of ^11^C-Sar into bile and normalize hepatocyte BA content.^48,49^ Some canalicular transporters, such as the export pump MRP2, are severely downregulated in response to cholestatic (eg, inflammatory) liver injury,^50,51^ whereas BSEP expression can be more durable and maintained in acquired cholestasis.^41,52^ The secondary changes in transporter expression can be explained by transcriptional induction or repression via regulatory nuclear receptor networks, including FXR and SHP, PXR, CAR,^53^ or inflammation-mediated interference with key transcription factors.^51,54^ In addition to transcriptional effects, posttranscriptional and posttranslational changes such as altered transporter protein localization or retention in subapical endosomal compartments, changes in expression or function of tight junction and tethering proteins, and changes in canalicular architecture and contractility may contribute to impaired hepatobiliary transport function in cholestasis.^23,30^

In addition to the liver, BA transport is altered in other tissues important for BA cycling in patients with cholestatic liver disease. In the small intestine, the consensus is that BAs negatively regulate IBAT expression and ileal BA absorption.^55,56^ As such, under normal physiological conditions, ileal BA absorption is homeostatically controlled to balance hepatic BA secretion, thereby protecting the hepatocyte from receiving a BA load that exceeds the canalicular transport capacity. In patients with cholestatic liver disease, much less is known regarding the changes in ileal IBAT expression and intestinal BA transport capacity. However, it has been reported that ileal conservation of BAs (as measured using the BA tracer ^75^SeCAT) is unchanged^57^ or increased^58,59^ in cholestatic patients. In patients with cholestatic liver disease, BAs and BA metabolites are significantly increased in the systemic circulation and urine. For the kidney, results from animal model studies generally supported the concept that IBAT expression and tubule BA absorption are decreased in response to the increased BA exposure. However the changes in kidney BA reabsorption or IBAT expression appear to be variable between different cholestatic models and between species, with fractional tubular taurocholate reabsorption decreasing from 93.1% to 89.5% in bile duct-ligated rats,^60^ and a range of reported cholestasis-associated decreases in kidney IBAT expression or activity in rats (decreased ~40% to 60%^60,61^) and wildtype mice (decreased ~0% to 55%;^62–67^). Moreover, it is difficult to reconcile the concept that kidney IBAT expression or activity is significantly decreased with the observation that the urinary loss of sulfated BAs (a poor IBAT substrate) versus non-sulfated BAs does not reflect their relative abundance in the plasma of cholestatic patients. Although there is a paucity of data on kidney IBAT expression in patients with liver or kidney disease, kidney IBAT mRNA and protein expression in biopsies collected from patients with chronic renal failure was similar to that of control subjects^68^ and a recent immuno-histochemical analyses of a limited number of kidney biopsies from cholestatic patients documented IBAT protein expression levels that were similar in liver disease subjects with or without cholemic nephropathy.^67^ These findings support the concept of persistent, ongoing IBAT-mediated reabsorption of conjugated BA from the renal proximal tubules in cholestatic patients.

Impaired BA homeostasis may also contribute to the pathogenesis and progression of metabolic dysfunction–associated steatotic liver disease (MASLD) and metabolic dysfunction–associated steatohepatitis (MASH)^69,70^ as reflected by observed elevated serum BA levels, and changes in BA composition with an increase in primary and conjugated serum BAs, which both have been linked to the severity of MASH and fibrosis.^71,72^ In addition, FXR–FGF19 feedback signaling is altered in MASH as evidenced by increased hepatic *CYP7A1* expression and serum C4 levels, and reduced serum FGF19 levels.^73,74^ Impaired FXR activation in MASLD could be explained in part by increased free fatty acids interfering with FXR-mediated activation of SHP, thus promoting BA synthesis and uptake through upregulated CYP7A1 and NTCP.^75^ Moreover, insulin resistance may also contribute to dysregulated BA synthesis and increased serum BA levels in MASLD/MASH.^69^ Impaired expression and function of hepatobiliary transporters, such as downregulation of canalicular Mrp2 observed in rodent models^76,77^ may contribute to cholestasis in MASLD. Recently, 3D digital reconstructions and computational simulations indicated increased pericentral biliary pressure and microcholestasis in patients with MASLD, which correlated with elevated cholestatic liver enzymes.^78^ Notably, increased periportal but decreased pericentral BSEP staining in liver biopsies from patients with MASH has been reported,^79^ which would also be consistent with the concept of pericentral microcholestasis in MASLD/MASH.^70^ In a preclinical model of diet-induced MASH, IBAT inhibition markedly improved glucose metabolism and histopathology.^80^ Biochemically, IBAT inhibition changed the retained BA composition to be more FXR agonistic, while markedly reducing hepatic retention of oxysterols and triglycerides. Together, the interruption of BA flux with IBAT inhibition led to multiple molecular consequences in the liver that are consistent with alleviating microcholestasis. These findings suggest that targeting transporter function and the BA circulation may be relevant not only for the treatment of cholestatic liver disease, but also metabolic liver diseases, which may also have a (micro)cholestatic component.^69,70^

## THERAPEUTIC TARGETING OF BA TRANSPORT IN CHOLESTATIC AND METABOLIC LIVER DISEASES— GENERAL REMARKS

From a therapeutic perspective, primary—mostly genetic—defects may be the target for positive functional modulators or chaperones depending on the molecular basis of the variant. In general, single-nucleotide changes that create an amino acid substitution, alter RNA stability, or disrupt exon splicing affect protein expression and function. The best example to date of therapeutic modulation of a genetic disease resulting from altered membrane transporter function does not come from hepatology, but pulmonology, where small molecules have proven effective in partially correcting the impaired Cystic Fibrosis Transmembrane Conductance Regulator (CFTR; *ABCC7*) activity caused by missense mutations in the *CFTR* gene.^81^ The use of comprehensive chemical libraries for screening and iterative creative medicinal chemistry led to the discovery and development of molecular chaperones that stabilize mutant CFTR misfolded proteins, promote sufficient membrane insertion and/or function, and impact patient outcomes. The clinical use of combinations of small-molecule modulators targeting the functional defects in different mutant CFTR protein variants significantly improved lung function, and as a result, the lives and life expectancies have markedly improved for many patients with cystic fibrosis (CF).^82^ As “proof of concept,” this remarkable therapeutic response to molecular modulation of a dysfunctional transporter (CFTR) suggests that a similar approach may have clinical benefit in patients with cholestasis associated with variant hepatobiliary transporters. Indeed, results reported for cell culture models of clinically relevant ABCB11 and ABCB4 variants have been promising,^83–85^ although these findings have not yet been translated to the clinic. A related strategy is the use of 4-phenylbutyrate (4-PB) or glycerol 4-PB as a general stabilizer of missense variants. This approach has shown functional improvement for mutant forms of BSEP in cell culture and clinical improvements in children with ABCB11 disease (PFIC2).^86^ These drugs are approved for use in children with select urea cycle defects and therefore may be considered for certain patients and gene variants. Another approach that may be beneficial in patients with specific primary genetic forms of cholestasis is to stimulate expression of hepatobiliary transporters using strategies such as FXR agonists. For example, transcriptionally enhancing expression of a defective transporter with partial activity, if the defective protein reaches the (canalicular) membrane, may reduce the hepatocellular burden of retained cholephiles such as BAs and improve liver function.

For ursodeoxycholic acid (UDCA), a mainstay anticholestatic drug that has been in modern clinical use for almost 3 decades, the mechanisms of action are mediated mostly at the posttranscriptional level through activation of protein kinase C, mitogen-activated protein kinases, and α5β1 integrins to enhance vesicular targeting of transporters to the cell membrane, thereby restoring hepatobiliary excretory function (reviewed in detail elsewhere).^87,88^ Conversely, regulatory transcriptional networks which control the expression of transporters and key metabolic enzymes can be targeted by pharmacological ligands for nuclear receptors such as FXR and various PPAR isoforms.^89,90^ Increased intrahepatic BA concentrations are a key driver of cholestatic liver injury^31^ and can be reduced by therapeutic activation of nuclear receptors to transcriptionally repress expression of CYP7A1, the rate-limiting BA biosynthetic enzyme, or to induce expression of endogenous secondary adaptive mechanisms for phase I/II detoxification and phase III elimination of BAs and other cholephiles. However, it should be noted that BA synthesis may already be maximally repressed in cases of severe cholestasis, as evidenced by the near complete suppression of *CYP7A1* in patients with advanced primary sclerosing cholangitis (PSC),^91^ and persistent enteronephrohepatic cycling may continue returning cytotoxic BAs to the cholestatic liver. Under those conditions, another therapeutic strategy that may show greater efficacy is the disruption of BA return to the liver by pharmacological inhibition of IBAT or NTCP. As a variation on that approach, severely cholestatic patients may benefit from systemic IBAT/ASBT inhibitors that target the “nephrohepatic” arm of the BA circulation under conditions when hepatobiliary BA secretion is severely impaired and enterohepatic BA cycling (targeted by “conventional” IBAT inhibitors) is minimal.

## TARGETING THE BA CIRCULATION TO TREAT LIVER DISEASE

### Early development of enterohepatic BA cycling blockers

Efforts to identify and clinically develop IBAT inhibitors as an orally available small-molecule enterohepatic BA cycling blocker date back almost 3 decades.^92^ The initial focus was to identify orally administered gut-restricted IBAT inhibitors as an effective replacement for BA sequestrants or ileal bypass, which, before the development of the statins, had been among the few available treatments for hypercholesterolemia-associated cardiovascular disease.^93,94^ Although BA sequestrants were safe, the combination of common gastrointestinal side effects and requirement for high doses (15–30 g/day) of the first-generation BA sequestrants reduced patient compliance and overall efficacy of these agents. Considering IBAT’s function as the primary mechanism for intestinal active BA absorption and its cell surface expression at the ileal brush border membrane, nonabsorbable inhibitors represented a potentially tractable alternative to sequestrants (Figure 2). Blocking intestinal BA reabsorption stimulates hepatic cholesterol demand for de novo BA synthesis, which is met by increasing hepatic clearance of plasma LDL and increasing hepatic cholesterol synthesis.^95^ Through the use of high-throughput screening and medicinal chemistry approaches, a series of high-affinity and selective IBAT inhibitors were identified and subsequently shown to be effective at reducing plasma LDL cholesterol levels and/or atherosclerosis in preclinical models.^96–98^ Although several IBAT inhibitors entered the clinical trial phase for treatment of hypercholesterolemia, development did not progress further, potentially due to concerns over dose-related diarrhea and efficacy when compared to newer classes of lipid-lowering agents such as higher potency statins and cholesterol absorption inhibitors.^99^ Several of the first IBAT inhibitors identified were absorbable and had considerable systemic exposure.^100,101^ However, subsequent efforts focused on developing oral drugs with low intestinal absorption to reduce potential adverse side effects related to drug metabolism or drug-drug interactions in the liver.^102^ The early pharmaceutical development of IBAT inhibitors before 2010^103^ and the medicinal chemistry approaches used to restrict gut absorption of IBAT inhibitors^104^ have been reviewed previously.

### Efficacy of enterohepatic BA cycling blockers for liver disease

#### IBAT inhibitors for MASLD/MASH

In addition to lowering plasma cholesterol levels, the first-generation BA sequestrant cholestyramine had been reported to improve glycemic control in a selected group of patients with type 2 diabetes.^105^ These findings were confirmed in later clinical trials for a second-generation BA sequestrant, colesevelam hydrochloride,^106^ which was approved in 2008 by the U.S. Food and Drug Administration (FDA) as adjunct therapy for diabetes.^107^ Mechanistically, BA sequestrants are thought to improve glycemic control by promoting BA stimulation of gut GLP-1 secretion,^108,109^ and through modulation of other BA-regulated pathways.^110^ Coinciding with an increased recognition of the metabolic actions of BAs,^111^ the clinical observations for BA sequestrants stimulated interest in gut-restricted IBAT inhibitors for the treatment of diabetes^112,113^ as well as MASLD/MASH.^69^ In preclinical models of diabetes and MASLD/MASH, IBAT inhibitors were effective at improving glycemic control and reducing hepatic steatosis and inflammation.^80,114–116^ However, in small studies of patients with type 2 diabetes, IBAT inhibitor effects on glycemic control were modest,^117–119^ and a larger phase II dose-finding IBAT inhibitor study found no significant liver-related clinical benefit in patients with hepatic steatosis and MASH without cirrhosis.^120^ Although disappointing as monotherapy, the findings do not exclude a possible role for gut-restricted IBAT inhibitors in combination with other therapeutic approaches, particularly for metabolic liver diseases, which may have a (micro)cholestatic component (discussed above).

#### IBAT inhibitors for cholestatic liver disease

The use of IBAT inhibitors for the treatment of pediatric cholestatic liver disease has been reviewed recently.^5^ Whereas a variety of genetic, immune, and environmental factors initiate cholestatic liver disease,^31,32,121^ retained BAs have been implicated as a common contributor to disease pathogenesis and progression.^1,2,3,4^ Based on their ability to redirect the BA circulation, gut-restricted IBAT inhibitors have been postulated to act in part by decreasing hepatic BA accumulation.^122–124^ It should be noted that this mechanism is not unique to IBAT inhibitors. NTCP inhibitors,^125^ FXR agonists,^49,126^ therapeutic BAs/BA analogs such as UDCA^127^ and norucholic acid (NCA; also called nor-ursodeoxycholic acid)^4,18,90,128^ are also thought to act in part by decreasing the mass and/or cytotoxicity of the liver BA pool. Before the advent of medical therapy for pediatric cholestatic liver disease, surgical biliary diversion had been shown to decrease circulating BAs and improve symptoms such as pruritus in progressive familial intrahepatic cholestasis (PFIC) and Alagille syndrome (ALGS) patients.^129,130^ Surgical intervention was not effective in all patients,^131^ but those who responded with a normalization of serum BAs achieved good long-term outcomes with little progression of cholestasis.^132^ Although there was some early pharmaceutical industry interest in the late 1990s in gut-restricted IBAT inhibitors as medical therapy to mimic the actions of surgical biliary diversion, clinical development for cholestasis would not begin in earnest until more than a decade later, potentially due in part to concerns over whether cholestatic liver disease would be a commercially viable market.^59^ Gut-restricted IBAT inhibitors have shown clinical benefit, such as reduction in pruritus in pediatric cholestatic patients^133–137^ and are approved as nonsurgical treatments for children with PFIC and ALGS.^138,139^ Underscoring the enormous challenges associated with translating promising targets to medical therapies for cholestatic patients, IBAT inhibitors are one of the few drugs approved by the FDA and EMA (European Medicines Agency) for treatment of cholestatic liver disease over the past 30 years, along with UDCA for primary biliary cholangitis (PBC) in 1996, the FXR agonist obeticholic acid for PBC in 2016, and most recently the PPAR agonists for PBC in 2024.

The mechanisms of action of the gut-restricted IBAT inhibitors have been explored in preclinical models of cholestatic liver disease, including 3,5-diethoxycarbonyl-1,4-dihydrocollidine (DDC) diet-fed mice,^140^ Mdr2(Abcb4)^−/−^ mice,^141,142^ and Cyp2c70^−/−^ mice.^143–145^ Classically, hydrophobic BAs are thought to damage hepatocytes through direct cytolytic or detergent effects at the ER and mitochondrial membrane and/or by inducing cell death pathways.^146^ In addition, more recent evidence indicates that BAs damage the liver by stimulating an inflammatory response under cholestatic conditions.^147,148^ In cholestatic mouse models, gut-restricted IBAT inhibitors effectively reduced the intrahepatic BA content and decreased parameters of liver injury, including ALT and AST, expression of genes involved in inflammation and fibrosis, and histological markers of fibrosis, macrophage infiltration, and the ductular reaction. These improvements were observed despite an increase in the overall hydrophobicity of the total or hepatic BA pool. Inhibition of IBAT blocks small intestinal absorption, redirecting BAs into the cecum and colon, where they can undergo microbial metabolism. The resultant unconjugated and secondary BAs are passively absorbed and travel back to the liver for uptake, conjugation, and secretion into bile. In mice, a compositional shift toward secondary BAs is a consistent finding with short-term IBAT inhibition and is particularly evident with long-term pharmacologic IBAT inhibition or IBAT genetic knockout.^80,149^ In PFIC patients, reduction in total serum BAs and shift in BA composition toward unconjugated BA species and a lower cholic acid/chenodeoxycholic acid ratio is a consistent finding in individuals who respond to administration of IBAT inhibitors.^150,151^ However, in contrast to mouse models, the shift in serum BA composition toward hydrophobic secondary BAs is more variable, which may reflect differences in colonic transit or levels of gut bacteria with BA dehydroxylating activity. Increasing evidence suggests that there may be an intrahepatic BA threshold for cholestatic liver injury, even for the hydrophobic BA pool characteristically found in humans, and that monotherapy or combination therapy that reduces liver BA residence may have benefit.^143,145,152^ An important question regarding the mechanism of action of the gut-restricted IBAT inhibitors is how changes in BA levels and BA composition translate to improvements in the cholestatic liver, particularly regarding modulation of inflammation and immunity.^121,153^ For example, in Mdr2^−/−^ mice, treatment with an IBAT inhibitor was associated with a significant reduction in hepatic CD11b+F4/80+ Kupffer cells and CD11b+Gr1+ neutrophils, expansion of anti-inflammatory Ly6C− monocytes, and decrease in pro-inflammatory/pro-fibrogenic Ly6C+ monocytes.^141^ Finally, it is important to note that factors in addition to BAs play critical mechanistic roles in the pathogenesis and progression of cholestatic liver disease, and interventions that selectively target myeloid cells or cholangiocytes in mouse models have demonstrated therapeutic benefits without reducing hepatic BA levels or BA pool hydrophobicity.^121,154^

#### Challenges for gut-restricted IBAT inhibitors

*Safety and tolerability:* The gut-restricted IBAT inhibitors have been generally well tolerated in children, with the predicted gastrointestinal effects (primarily diarrhea and abdominal pain) constituting the most common treatment adverse events.^5,155^ Unexpected for a gut-restricted compound, elevations in liver enzymes have been observed in some PFIC and Alagille patients administered IBAT inhibitors. The mechanism is unclear and is unlikely due to direct hepatic toxicity of the poorly absorbed IBAT inhibitor. Several different BA-related mechanisms come to mind. In mice that primarily use taurine for BA conjugation, administration of a BA sequestrant has been reported to deplete the free cysteine pool, which serves as a common precursor for taurine and glutathione biosynthesis. This reduces hepatic glutathione synthesis and increases oxidative stress under conditions where hepatic BA synthesis is greatly elevated.^156^ However, it is unclear if this mechanism would be operative in humans. In contrast to mice, ~70% of the newly synthesized BAs in humans are conjugated to glycine rather than taurine, and the hepatic BA biosynthetic rate is significantly lower in humans versus mice.^157^ Although transient elevations in liver enzymes have been observed in patients taking BA sequestrants,^158^ the transaminase increases are mild and resolve without stopping therapy. Another potential mechanism is toxicity associated with the increased BA pool hydrophobicity observed with IBAT inhibitor treatment.^143,150^ That potential mechanism is indirectly supported by studies in a preclinical dog model of an FGFR4 inhibitor, which significantly increases hepatic BA synthesis. Administration of the FGFR4 inhibitor increased serum aminotransferase, and the elevation in ALT correlated with serum levels of hydrophobic secondary BAs.^159^

*Efficacy:* With regard to markers for surgical diversion (partial external biliary diversion) and IBAT inhibitor efficacy, reductions in serum total BA levels are associated with decreased pruritus and the potential for improved event-free and transplant-free survival in Alagille syndrome and PFIC patients.^5,130,133,135,137^ However, fecal BAs may also be increased after administration of IBAT inhibitors up to a theoretical level measured in subjects with inherited IBAT (*SLC10A2*) deficiency.^160,161^ For example, in a phase 1 study, administration of an IBAT inhibitor once daily significantly increased fecal BA excretion (as measured using a 24 h fecal collection) by ~1.6–3.2-fold in healthy adults and ~8-fold in adult patients with type 2 diabetes.^117^ Although tracking fecal BA levels may have a role in monitoring IBAT inhibitor efficacy, in practice, measurements of total and primary BAs in 48-hour fecal collections or single stool samples are used primarily for diagnosing BA diarrhea as a cause of chronic diarrhea.^162,163^ More widespread adoption of fecal BA measurements is currently impeded by limited availability of sample preparation/LC–MS/MS analysis for clinical care^164^ and the long fecal sample collection periods (24–48 h) required to ensure accuracy and account for the normal diurnal rhythm for BA secretion^165^ or the impact of diet or liver disease on fecal total BA excretion or composition.^166^ However, fasting plasma levels of the BA biosynthetic intermediate 7-alpha-hydroxy-4-cholesten-3-one (C4) are a biomarker currently used clinically for the diagnosis of BA diarrhea. C4 has been measured in clinical trials of IBAT inhibitors and has been reported to correlate with fecal bile levels in patients with PSC.^166^ As such, C4 may have utility in optimizing dosing of the different IBAT inhibitors or monitoring IBAT inhibition.^167^

A significant potential limitation is that the efficacy of gut-restricted IBAT inhibitors in patients with a severe block in BA secretion, such as patients with inherited protein-truncating mutations in BSEP or complete absence of BSEP protein. Patients with BSEP variants that induce nonfunctional protein show a significantly reduced probability of survival with native liver.^168^ Moreover, those patients respond poorly to surgical interruption of the BA enterohepatic circulation.^169,170^ Gut-restricted IBAT inhibitors were designed in part to medically recapitulate surgical approaches such as partial external biliary diversion, and thus, patients with a severe block in BA secretion are likewise not predicted to benefit from medical therapy acting at the intestinal level to block BA return to the liver.^136^ However, the reasons for the lack of efficacy are still unclear. One potential reason is related to the concept of a hepatic threshold for pathological accumulation and retention of hydrophobic/cytotoxic BAs.^145,146^ Although data on the BA content of normal and cholestatic human liver is limited, the total BA content was found to be elevated in infants with biliary atresia^171^ and in patients with other forms of cholestatic liver disease.^172,173^ Studies using the HepaRG hepatocytes reported that cellular hydrophobic BA accumulation predicted the potential for drug-induced cholestasis.^174^ In a preclinical model (Cyp2c70^−/−^) with a humanized hydrophobic BA composition, hepatic hydrophobic BA accumulation correlated with cholestatic liver injury and therapeutic response.^145^ Under conditions of significantly impaired canalicular BA export, alternative strategies to reduce hepatic BA synthesis or accretion may be beneficial, and these patients may be candidates for interventions that target nephrohepatic BA cycling.^5^

## POTENTIAL FOR SYSTEMIC IBAT/ASBT INHIBITORS AND DUAL ASBT/NTCP INHIBITORS

BAs are cytotoxic at high free concentration, and interventions that shunt BAs away from the liver appear to slow or even reverse disease progression. The gut-restricted IBAT inhibitors block only part of the return of BAs to the liver. Notably, it does not block ongoing IBAT-mediated reabsorption of BAs from the kidney or hepatocyte NTCP-mediated BA re-uptake from blood in cholestatic patients with elevated serum BA levels. However, systemic inhibitors that block IBAT-mediated BA reclamation in the ileum and kidney (Figure 2) may be more effective at redirecting BAs away from the cholestatic liver for elimination in feces or urine.^19^ There are several barriers to pursuing this potential therapeutic strategy. Since the IBAT inhibitors developed and approved to date are poorly absorbed and largely gut-restricted, different strategies are required to design/develop an absorbable small-molecule IBAT inhibitor with acceptable bioavailability, pharmacokinetics, renal tubule IBAT target engagement, drug metabolism, and safety properties. Regarding efficacy, better estimates of the mass of non-sulfated/non-glucuronidated BAs that undergo glomerular filtration and reabsorption by the proximal tubules in cholestatic patients are needed.^175–178^ This would provide critical information regarding the amount of BA that can be potentially eliminated via this route each day, acknowledging that glomerular BA filtration will decrease as serum BA levels are reduced. However, one of the most significant potential hurdles is the risk that increasing BA elimination in urine will induce acute kidney injury (cholemic nephropathy).^179,180^

The term Cholemic Nephropathy (also called bile cast nephropathy) describes the renal dysfunction/kidney injury found in patients with obstructive cholestasis, and other forms of liver disease, ranging from drug-induced cholestatic liver injury to PSC to liver cirrhosis.^181–183^ Systemic hemodynamic alterations associated with liver disease are thought to play a major pathophysiologic role in development of the renal dysfunction,^184^ however non-hemodynamic mechanisms such as BA-induced renal tubular injury have also been implicated in the pathogenesis^180,181,185–188^ A previous study using bile duct-ligated (BDL) mice provided evidence that urinary elimination of potentially toxic BAs contributed to kidney injury by damaging the distal collecting ducts, thereby initiating a cascade leading to bile cast formation, progressive interstitial nephritis, and tubulointerstitial renal fibrosis.^179^ FXR knockout mice with a hydrophilic BA pool or administration of the BA analog norucholic acid were protective against the development of cholemic nephropathy in this model.^179,189^ Although compelling, a recent study using new high-resolution approaches such as intravital microscopy to directly observe glomerular filtration and tubular BA reabsorption and a systemically bioavailable IBAT inhibitor in BDL mice has revisited the underlying mechanisms responsible for cholemic nephropathy.^67^ The findings from that study, which included Cyp2c70^−/−^ mice with a hydrophobic and more human-like BA composition, directly challenge the previous paradigm. The results strongly support a model whereby cholemic nephropathy is initiated by elevated BAs damaging the IBAT-expressing proximal tubule epithelium, and that inhibition of renal IBAT to block BA uptake by the proximal tubule epithelium and divert BAs into urine is protective for the nephron. Key findings from that study support the following working model for the development and progression of cholemic nephropathy: (1) IBAT-mediated BA uptake/enrichment in proximal tubule epithelial cells induces oxidative stress/cell death and is an early initiating event. (2) Tubule cell debris sloughed into the proximal tubule lumen moves downstream and is concentrated in distal tubules and collecting ducts to induce cast formation, tubule dilatation, and disrupt membrane integrity. (3) Leakage of BAs into the renal interstitial space damaged the peritubular capillary endothelium and compromised barrier function. (4) Leakage of blood and primary urine into the renal parenchyma induces a tubulointerstitial nephritis and progressive renal fibrosis.^67^ Of importance regarding the therapeutic potential of a systemic IBAT inhibitor, the findings in this preclinical model suggest that redirecting BAs from the cholestatic liver via the renal tubules for elimination in urine may be renoprotective rather than promote cholemic nephropathy. The relationship between liver disease and acute kidney injury and the role of renal IBAT and BAs in the pathogenesis of cholemic nephropathy has been reviewed recently.^190^

A variation on this strategy is the development of systemically bioavailable small molecules that simultaneously inhibit both the IBAT and structurally related NTCP (Figure 2). Simultaneously targeting both carriers may be more effective in directing BAs away from the cholestatic liver into urine for excretion from the body.^191^ As such, even in the cholestatic patient population who exhibit a total block in biliary BA secretion, an absorbable mixed IBAT/NTCP inhibitor strategy is predicted to reduce the hepatic burden of cytotoxic BAs and potentially yield therapeutic benefit.

## NTCP INHIBITORS

The distinct therapeutic, clinical, and research worlds of BA metabolism and viral hepatitis unexpectedly converged in 2014 when Yan et al^192^ discovered that the membrane receptor for HBV is NTCP. Further confirmation that the HBV pre-S1 surface domain directly interacts with NTCP (*SLC10A1*) came from Ni et al,^193^ who reported that the HBV-derived surface peptide myrcludex B (bulevirtide) interferes with HBV cellular entry by binding to the hepatocyte basolateral surface via NTCP. These seminal and intriguing findings launched novel and relevant molecular, physiologic, and clinical studies on the potential reciprocal effects of BA flux on HBV entry and HBV docking peptide effects on conjugated BA import through NTCP. Moreover, these studies solidified the role of NTCP as the primary receptor for HBV and primary mechanism for conjugated BA import and revealed the therapeutic potential of blocking NTCP function for both viral hepatitis and cholestasis (Figure 2).

Studies of bulevirtide’s efficacy as an anti-HDV therapeutic agent^194,195^ provided evidence that the viral entry inhibitor is a potent blocker of conjugated BA import into hepatocytes.^196–199^ For example, Wedemeyer et al reported a dose-dependent elevation of serum conjugated BAs to levels averaging ~30 μM in participants who received the 2 mg dose, and ~60 μM in response to the 10 mg weekly dose.^195^ These serum BA findings from pharmacologic inhibition of NTCP with bulevirtide align with those from reports of patients with absent or impaired NTCP expression due to NTCP missense mutations, which in subjects with the most severe mutations, can lead to serum levels of BAs >1 mM.^200,201^ It is intriguing to note that pruritus was not a feature of patients with severe NTCP gene mutations, yet in clinical trials, more patients on bulevirtide reported pruritus (14%) compared to placebo (0%). Of note, the IC_50_ of bulevirtide for blocking viral entry is several hundred-fold less than that for inhibition of conjugated BA transport, suggesting that the dosage needed clinically to inhibit HBV infection may not fully inhibit hepatic BA uptake. Dose optimization for NTCP blockers will be key for antiviral drug development (including combinations) and may also inform inter-personal differences in the response to treatments, especially among those with NTCP gene variants. Teasing apart the importance of NTCP protein expression levels in the livers of patients with HBV and varying hepatopathies from any cause will require well-designed studies.

Building upon the evidence that an agent that impairs human NTCP function can interrupt HBV docking and reduce the import of conjugated BAs into the liver provides an intriguing new level of attention to small-molecule inhibition of NTCP as both an antiviral and anticholestatic target.^202^ With the success and approval of intestinal IBAT inhibitors as antipruritic agents in genetic forms of cholestasis (mainly ALGS and several genetic forms of intrahepatic cholestasis [PFICs]), it is notable that not every genetic cholestatic patient responds to intestinal IBAT inhibition.^5^ It is therefore clinically relevant to explore additional means to reduce ongoing BA loading of the cholestatic liver, which includes further inhibiting NTCP-mediated BA uptake beyond that mediated by the well-described transcriptional and posttranscriptional adaptive mechanisms that reduce NTCP expression and function. Investigation of Ntcp inhibition alone in preclinical cholestatic models is limited, since other hepatocyte BA transporters (ie, OATP family members) in mice can compensate for impaired Ntcp expression.^42,157,203^ Elimination of non-Ntcp BA transport is a potential way forward, as well as the utilization of the humanized hepatocyte liver re-population model.^204^ Both approaches have utility and gaps (eg, immunodeficiency in the humanized model) to provide a means to explore NTCP/Ntcp inhibition with scientific rigor and appropriate controls. Notably, Ntcp blockade attenuates a BA-induced cytokine-induced inflammatory response in hepatocytes, which otherwise contributes to the propagation of cholestatic liver injury.^147^ In line, Ntcp inhibition by myrcludex B has demonstrated hepatoprotective effects in mouse models of cholestasis, by reducing BA load in cholestatic hepatocytes and increasing the biliary phospholipid (PL)/BA ratio at the bile duct level (protecting bile ducts).^125^ Of interest, Ntcp inhibition by myrcludex B (bulevirtide) worsened weight loss in the mouse BDL model, while exacerbating liver pathology in the Mdr2(Abcb4)^−/−^ mouse,^125^ suggesting additional explorations are needed to characterize the consequences of long-term Ntcp inhibition, especially in cholestasis. Despite these experimental caveats, there are multiple attractive features of NTCP/Ntcp inhibition, including the potential to block residual NTCP activity that persists despite adaptive mechanism to reduce NTCP expression/activity, the apparent absence of adverse consequences associated with genetic interruption of NTCP function in human subjects, and unloading the cholestatic hepatocyte is likely to require a multi-pronged approach to tailor the array of therapeutic approaches to the individual patient’s underlying defect and degree of cholestasis (see the Summary section). The expanding landscape of NTCP inhibitors for the treatment of liver diseases has recently been reviewed.^205^

## NUCLEAR RECEPTOR MODULATORS AND RELATED AGENTS

As the central regulator of BA homeostasis, FXR is an attractive therapeutic target in cholestasis since its pharmacological stimulation limits intrahepatic BA levels by downregulating hepatocellular uptake via NTCP and BA synthesis via CYP7A1, while stimulating expression of canalicular (BSEP) and basolateral (OSTα/β, MRP4) efflux systems.^90,206–208^ Moreover, stimulation of FXR also has anti-inflammatory and anti-fibrotic effects.^121,209,210^ Potential limitations, including the already pre-existing stimulation of these pathways by retained BAs in cholestasis and impairment of FXR signaling by (post)transcriptional changes,^41^ may be overcome by the very high affinity of therapeutic FXR ligands. However, recent data suggest that BA synthesis may already be maximally repressed in advanced cholestasis and therefore may no longer be amenable to further pharmacological suppression via FXR.^91^

In 2016, the FXR agonist obeticholic acid (OCA; 6-ethyl-chenodeoxycholic acid) was conditionally approved as a second-line agent for primary biliary cholangitis (based on NCT01473524^211^). Subsequently, after negative Phase IV data (COBALT NCT02308111^212^) and analyses by regulatory agencies in both Europe and the United States, serious safety signals and concerns about overall efficacy were identified that led to a recommendation for withdrawal from the European market in 2024 (https://www.ema.europa.eu/en/news/ema-recommends-revoking-conditional-marketing-authorisation-ocaliva). In parallel, reviews by the FDA have led to serial updates in prescribing information over a period from 2021 (concerns that serious liver injury from obeticholic acid can occur in patients with advanced cirrhosis) to December 2024 (concerns that serious liver injury from obeticholic acid can also occur in patients without cirrhosis) (https://www.fda.gov/drugs/drug-safety-and-availability/serious-liver-injury-being-observed-patients-without-cirrhosis-taking-ocaliva-obeticholic-acid-treat). In addition to the unfavorable safety–efficacy relationship for OCA, inherent problems in the study methodology including challenges for confirmatory trials to retain trial participants on placebo following conditional approval of drugs) and new (rekindled) questions arising around the relationship between surrogate parameters, such as improvement of ALP and long-term clinical outcomes, have added to the current concerns regarding the use of OCA in PBC.

Notably, one key side effect of OCA, and common to all potent FXR agonists,^213^ is worsening of pruritus^214^ regardless of the underlying disease under study. Together with the observation that NTCP-deficient patients have elevated serum BAs but are not pruritic, these finding for the FXR agonists suggests that the cholestatic pruritogen (or pruritogens) is a molecular entity arising from the intra-hepatocytic FXR response (eg, IL-31).^215^ Also of concern, but generally addressable with adapted targeted therapies, is an elevation in plasma LDL cholesterol levels in response to OCA, first detected in trials of patients with MASH^216^ but also observed in PBC.^211^ Similar side effect profiles with modest, principally biochemical, responses to non-steroidal FXR agonists cilofexor and tropifexor in cholestatic disorders or MASH have been reported.^217–222^ Whether or not there are similar serious safety concerns with non-steroidal FXR agonists as have been reported for the BA-based OCA is an important question that remains to be answered. Indeed, as a BA analog and BA transporter substrate, OCA (6-ethyl-chenodeoxycholic acid) is conjugated to taurine or glycine and retained in the BA circulation like native hydrophobic BA species. Part of the toxicity associated with 6-ethyl-CDCA could potentially be attributed to its inability to undergo efficient phase II sulfation,^223^ which plays an important role in the elimination of native hydrophobic BAs.^8^ Taken together, roles for FXR modulation in therapies for a variety of liver disorders are not clear and therefore mixed at the present time. There are efficacy signals as well as safety concerns that are both in need of additional data to better understand the roles of these agents for future therapies. There are certainly potential opportunities for exploration of FXR agonists in liver disorders, especially with appropriate patient selection and monitoring, and as a component of combinatorial therapies.^224^

PPAR agonists, often molecules that can target more than one member of the PPAR family, are showing significant promise in several cholestatic disorders. Some agents are not new (eg, fibrates targeting PPAR alpha), whereas others are investigational (saroglitazar, targeting PPARα/γ) or are recently approved (elafibranor targeting PPARα/γ; seladelpar targeting PPAR delta) as second-line therapy for PBC (recently reviewed^225^ and relevant studies^226–229^). Although there is direct modulation of BA metabolism by reducing BA synthesis (PPAR alpha and delta), increasing BA glucuronidation (PPAR alpha), and increasing canalicular BA and PL excretion (PPAR alpha),^230,231^ PPAR agents’ principal actions appear to be more anti-fibrotic and anti-inflammatory through non-parenchymal cells, properties known for many years.^232–236^ These promising studies, especially with an apparent tolerable safety profile provided to date, plus potential amelioration of pruritus and fatigue^237^ make these agents alone or in combination, drugs to be mindful of for metabolic and cholestatic liver diseases, as we accumulate more long-term treatment data. Notably, human FXR regulates PPAR alpha,^238^ providing additional rationale for combining these approaches in the treatment of cholestatic disorders.^239^

The FXR target gene FGF19 is highly upregulated by FXR agonists in both the ileum and liver, acting through a signaling cascade involving the FGFR4/β-klotho complex to markedly suppress BA synthesis.^240^ There are other non-hepatic targets for FGF19, indicating potential global metabolic effects as well as additional safety considerations.^241^ Recent studies in MASH and cholestasis of a modified FGF19 molecule (aldafermin), designed to minimize the protein’s mitogenic properties and enhance protein stability, provided mixed results regarding potential biochemical and histologic improvement.^242,243^ Notably, aldafermin showed potential anti-fibrotic effects by improving noninvasive markers of liver fibrosis (without reducing ALP as a biochemical marker of cholestasis),^244^ which was attributed to reductions in the hydrophobic (potentially pro-fibrogenic) BA profile.^245^ Although encouraging, it is premature to draw conclusions regarding the safety and efficacy of aldafermin in cholestatic or metabolic liver disorders. For a more detailed summary of the current clinical development of FXR and PPAR ligands and their downstream targets (FGF19 and FGF21), we refer to recent comprehensive reviews focused on this topic.^90,207^

## NORUCHOLIC ACID

Nor-ursodeoxycholic acid (norUDCA; recently named norucholic acid, NCA) is a side chain shortened derivative of UDCA which is resistant to conjugation with glycine and taurine and therefore undergoes cholehepatic shunting between cholangiocytes and hepatocytes, resulting in a bicarbonate-rich “hypercholeresis,” that is, the amount of bile flow exceeds the amount of (infused) BAs and can be explained by marked stimulation of bicarbonate secretion into bile.^246–248^ Despite the small biochemical difference lacking only a methyl group compared to UDCA, the physiological and therapeutic effects of NCA are profoundly different from UDCA.^249^ norUDCA was recently renamed as NCA to emphasize these unique mechanisms and avoid confusion with UDCA (WHO Drug Information, 2020, 34[4]). Intriguingly, further side chain modification by removing one additional methyl group (bisnorUDCA) results in loss of cholehepatic shunting, bicarbonate-rich hypercholeresis, and its associated therapeutic effects in preclinical mouse models.^250^

Notably, the concept of a bicarbonate-rich hypercholeresis induced by BAs was originally proposed as one of the key therapeutic mechanisms of action of UDCA by Serge Erlinger and his team based on observations in the isolated perfused liver.^251,252^ However, this UDCA effect is only observed after exhaustion of BA conjugation by depletion of hepatic taurine stores in the perfused liver ex vivo, but probably does not occur in vivo to a relevant degree. Subsequently, Alan Hofmann and coworkers demonstrated that NCA and other nor BAs, which are intrinsically resistant to conjugation independent from exhaustion of taurine and glycine stores in the liver, induce a bicarbonate-rich choleresis as a result of their capability to undergo “cholehepatic shunting.”^246,253,254^ Key steps of the cholehepatic shunt pathway for NCA or similar nor BAs include efficient hepatic extraction and secretion into bile, passive absorption in the biliary tract, and return to the liver via the periductular capillaries for uptake and resecretion into bile. The ability of NCA to evade hepatic conjugation to taurine and glycine and undergo multiple rounds of cholehepatic cycling before glucuronidation and elimination is likely critical for the superior choleretic activity of NCA. Conversely, exogenous UDCA is efficiently conjugated and competes with endogenous BAs for the limited carrier-mediated BA uptake in the biliary tract, thereby reducing cholehepatic shunting and directing UDCA into the enterohepatic cycle along with the endogenous conjugated BA pool.

NCA appears to induce a bicarbonate-rich choleresis by at least three different mechanisms. (1) NCA is actively secreted in its anionic form into bile, where it accepts a proton from carbonic acid in ductular bile, thereby generating a bicarbonate anion (HCO_3^–^_).^255^ The protonated NCA is sufficiently hydrophobic to undergo passive absorption by cholangiocytes, cross the biliary epithelium, and travel via the periductular capillary plexus back to the hepatocyte for uptake and resection into bile. Support for this model comes from recent findings demonstrating that the choleretic activity of NCA does not require ASBT, OSTαβ, or OATP1a/1b.^18^ (2) In addition to the ability of NCA to generate of a bicarbonate anion with each cholehepatic cycle, NCA can stimulate HCO_3^–^_ secretion via activation of the calcium-activated chloride channel transmembrane member 16A (TMEM16A, also named Anoctamin-1, ANO1), which stimulates bicarbonate secretion via coupling to the chloride/bicarbonate anion exchanger AE2.^18^ Notably, conjugated UDCA can also activate TMEM16A, but in contrast to NCA which enters cholangiocytes by passive diffusion, this effect requires active transport into cholangiocytes via ASBT.^256^ (3) NCA restores TGR5 expression on cholangiocytes (shown to be reduced in preclinical models and PSC livers), which may further amplify the stimulation of HCO_3^–^_ secretion.^257^

The synergistic therapeutic concepts of cholehepatic shunting (allowing bile duct targeting) and a bicarbonate-rich hypercholeresis to restore/reinforce the bicarbonate umbrella protecting bile ducts against BA toxicity^258^ encouraged the preclinical and clinical development of NCA to treat cholangiopathies such as PSC,^128,259^ PBC, and other cholestatic as well as metabolic liver disorders.^249,260^ Interestingly, non-BA compounds such as sulindac can also undergo cholehepatic shunting.^261^ Although sulindac has been shown to improve liver biochemistries in patients with PBC who responded incompletely to UDCA, this concept was not further pursued therapeutically, probably due to the lack of histological improvement as the gold standard at that time.^262^

In addition to stimulation of bicarbonate-rich choleresis, NCA showed anti-cholestatic, anti-apoptotic, anti-inflammatory, and anti-fibrotic effects in preclinical mouse models, most prominently the *Mdr2/Abcb4* ^*−/−*^ mouse model of sclerosing cholangitis,^128,250,263^ but also non-cholestatic models such as *Schistosoma mansoni* or lymphocytic choriomeningitis virus (LCMV) infection, among others.^264–266^ Notably, the immunomodulatory effects of NCA may help explain these broad therapeutic effects. NCA attenuated CD8^+^ T cell proliferation and related liver injury via mTOR inhibition.^265^ Recent findings demonstrated that NCA improves intestinal inflammation in various mouse models by targeting T_H_17 pathogenicity and trans-differentiation, suppresses T_H_17 effector function, and enriches Treg abundance in various mouse models of intestinal inflammation,^265^ potentially expanding the therapeutic mechanisms of NCA also to the gut (eg, PSC-IBD). NCA suppressed reactive phenotype activation and proliferation in human extrahepatic cholangiocyte organoids by suppressing sphingosine-1-phosphate receptor 2 (S1PR2) signaling.^267^ These intriguing therapeutic mechanisms have stimulated the clinical development of NCA in PSC and other liver pathologies such as PBC and MASLD/MASH.^249^ In line with beneficial preclinical effects in the *Mdr2/Abcb4* *^−/−^* mouse model of sclerosing cholangitis,^128^ a phase II trial demonstrated improvement of biochemical markers of cholestasis in PSC patients by NCA irrespective of prior exposure and response to UDCA^259^ and a phase III trial investigating NCA treatment in PSC patients showed positive results for the primary and key secondary endpoint (partial normalization of ALP and stabilization of histological disease stage) (NCT03872921).

Further potential applications for NCA includes the full spectrum of human ABCB4 deficiency (encouraged by the efficacy in the *Mdr2/Abcb4* *^−/−^* mouse model), CF (stimulation of CFTR-independent HCO_3^–^_ secretion via TMEM16A), PBC, non-anastomotic strictures after liver transplantation (also linked to impaired expression/function of MDR3/ABCB4) and MASLD (microcholestasis)^249^ with already ongoing phase II trials in PBC (EudraCT2021-001431-56) and MASH (NCT05083390).

## SUMMARY AND FUTURE OUTLOOK

We have arrived at a new era in the treatment of cholestatic disorders. This has been made possible by incorporating findings from discoveries into the molecular pathogenesis of cholestasis and adaptive processes that direct rational therapeutics to improve patients’ lives. One ready example is the success of IBAT inhibition to relieve intractable pruritus in PFIC and ALGS patients^5^ based upon knowledge that interruption of the enterohepatic circulation of BAs at the level of ileal reclamation can ameliorate hepatic BA retention. Another recent advance is the consideration of the relevant role of renal BA reclaimation and the enteronephrohepatic (rather than simply enterohepatic) circulation of BAs in health and disease. These paradigm not only better explains the whole body pharmacodynamics of the BA recirculation but also provide insights into new feasible targets for consideration to alter BA retention in the liver and other organs.

In addition to altering membrane transporter function by targeting ASBT and NTCP, there is an array of potentially additive therapeutic approaches based upon nuclear receptor-mediated transcriptional modification of BA synthesis and transport genes (FXR and PPAR targets), and the unique choleretic capacity of NCA and the cholehepatic shunt. Detailed clinical studies will not only help identify groups of patients with the various cholestatic disorders who may respond to these therapies, but also facilitate understanding and addressing some of the common side effects that can impair patient adherence (eg, diarrhea with IBAT inhibitors, lipid abnormalities, and pruritus with FXR agonists). With proper studies, roles for these newer agents may be found as monotherapy or in combination with UDCA and other emerging therapies, leading us to strongly believe that we are entering an era with multiple medical options to address the varied etiologies and consequences of cholestasis. Finally, although beyond the scope of this review, it should be noted that any therapeutic modulation of BA transport within the enterohepatic circulation will impact both microbial composition and microbial BA metabolism, including the exponentially expanding spectrum of microbial BA metabolites that may have a role in the regulation of metabolism and immunity (recently reviewed).^268^
